# Conversion surgery for gastric remnant cancer with liver metastasis after nivolumab combination chemotherapy achieving pathological complete response: a case report and literature review

**DOI:** 10.1186/s40792-024-01905-x

**Published:** 2024-05-01

**Authors:** Kaori Katsumata, Yosuke Morimoto, Junya Aoyama, Toru Yamada, Yusuke Katsuki, Ryo Nishiyama, Tomohisa Egawa

**Affiliations:** Department of Surgery, Saiseikai Yokohamashi Tobu Hospital, 3-6-1, Shimosueyoshi, Tsurumi-Ku, Yokohama, Kanagawa 230-0012 Japan

**Keywords:** Gastric cancer, First-line chemotherapy, Nivolumab combination chemotherapy, Pathological CR

## Abstract

**Background:**

Nivolumab combination chemotherapy has recently emerged as a potential first-line treatment for patients with unresectable or metastatic gastric cancer (GC). Further research has indicated that R0 resection by conversion surgery could be an effective treatment strategy to improve overall survival. However, there have been limited reports on the successful application of conversion surgery following combination chemotherapy achieving pathological complete response (pCR) in cases of advanced gastric remnant cancer with liver metastasis. Here, we present a case of long-term survival in a patient who underwent this treatment.

**Case presentation:**

A 54-year-old man was initially referred to our department for treatment of stage III (cT3N1M0) gastric cancer where he underwent laparoscopic distal gastrectomy and D2 lymph node dissection. After a year of uneventful follow-up, the patient was diagnosed with a tumor in the gastric remnant combined with liver metastasis, resulting in a diagnosis of stage IV (cT3N0M1) gastric remnant cancer. Subsequently, the patient was treated with four cycles of TS-1, Oxaliplatin, and Nivolumab as the first-line regimen. Remarkably, both the remnant tumor and liver metastasis exhibited significant shrinkage, and no new lesions were found. Given this response, conversion surgery was performed to achieve complete resection of the remnant gastric cancer and liver metastasis, followed by laparoscopic remnant gastrectomy and partial hepatectomy. Pathological examination revealed the absence of residual carcinoma cells and lymph node metastases. Postoperatively, the patient was treated with adjuvant chemotherapy with **S-1** for 1 year, and survived without recurrence for 18 months after conversion surgery.

**Conclusions:**

Nivolumab combination chemotherapy shows promise as a clinically beneficial treatment approach for gastric remnant cancer with liver metastasis, particularly when pCR can be achieved following conversion surgery.

## Background

As the third most common cause of cancer related deaths worldwide, gastric cancer (GC) presents a significant global health concern.[1] One of the reasons for this is that GC is often diagnosed at advanced stages, for which there are limited treatment options. Historically, first-line treatment options for GC primarily included cytotoxic chemotherapy regimens, such as fluoropyrimidines and platinum-based compounds. Despite their efficacy, in some cases, these treatments often result in a temporary response and limited survival benefits. The 5-year overall survival rate of patients with stage IV GC is only 5.7% [1].

Remnant GC has particularly poor prognosis. One of the reasons for this is its distinct pattern of lymphatic metastasis compared to primary gastric cancer, with a propensity for central lymph node involvement due to changes in lymphatic flow [2, 3]. This type of lymphatic metastasis, particularly towards the central lymph nodes, is associated with poor prognosis [4].

In recent years, immunotherapy, particularly immune checkpoint inhibitors (ICI), has emerged as a promising treatment strategy for GC. The introduction of ICI drugs, such as nivolumab, has transformed the therapeutic landscape of advanced GC. Nivolumab, a programmed death-1 (PD-1) inhibitor, acts by blocking the PD-1 receptor on T cells, thus preventing its interaction with PD-L1 and PD-L2 ligands on tumor cells. This blockade unleashes the cytotoxic activity of the immune system against cancer cells, potentially leading to durable responses [5].

A recent clinical trial, ATTRACTION-2 [6], was the first to demonstrate the efficacy of nivolumab as a third-line regimen for unresectable advanced GC and recurrent GC. Furthermore, the Checkmate649 [7] and ATTRACTION-4 [8] studies demonstrated the efficacy of nivolumab-based combination chemotherapy as a first-line regimen for advanced or metastatic gastric adenocarcinoma with HER2 negative. In Japan, the guidelines for GC have recommended this regimen since November 2021 [9]. However, the reports of stage IV GC patients achieving pathological complete response (pCR) with nivolumab combination chemotherapy published to date are limited to date.

Stage IV gastric cancer is a complicated condition in which peritoneal dissemination, hematogenous metastasis, and distant metastasis are intertwined; as such, it is difficult to consider using a unified criterion. To clarify treatment strategies, a subclassification of stage IV GC was proposed by Yoshida, et al. [10], termed the Yoshida classification, which classifies cases based on the presence or absence of peritoneal dissemination. Category 1 included resectable distant metastases, category 2 included unresectable distant metastases, category 3 included peritoneal dissemination, and category 4 included peritoneal dissemination and unresectable distant metastases. Categories 1 and 2 show relatively good prognoses compared with the other categories. An international multi-center collaboration retrospective study, the CONVO-GC-1 (Conversion Therapy for Stage IV Gastric Cancer) [11], was conducted to evaluate the overall survival for stage IV GC according to each category of Yoshida’s classification. This study showed that the median survival time (MST) of all patients who underwent resection was 36.7 months, and those of R0, R1, and R2 cases which underwent resection were 56.6, 25.8, and 21.7 months, respectively. Overall, this study concluded that R0 resection by conversion surgery could be an effective treatment strategy to improve overall survival in all categories of Yoshida’s classification.

Herein, we report a rare case of stage IV remnant GC with liver metastasis treated with nivolumab combination chemotherapy as the first-line regimen, in which we performed conversion surgery and achieved pCR.

## Case presentation

A 54-year-old man with no significant medical history, except for a 25-year history of smoking (10/day), presented with epigastric pain and sought medical attention at a clinic. Upper gastrointestinal endoscopy revealed an irregular ulcerative lesion, prompting a referral to our institution for further evaluation. Upper gastrointestinal endoscopy performed at our hospital revealed a type 2 tumor in the posterior wall of the lower gastric body. Biopsy confirmed the presence of adenocarcinoma. At the time of diagnosis, blood testing revealed a carcinoembryonic antigen (CEA) level of less than 1.0 and CA19-9 was under 0.1 U/ml, which indicated no elevation of tumor markers. Genetic tests, such as tumor mutation burden (TMB) and mismatch repair (MMR) genetic mutation were not conducted. Contrast-enhanced computed tomography (CT) demonstrated irregular wall thickening in the lower part of the gastric body with associated contrast enhancement. The tumor partially extended to the posterior aspect, and was accompanied by an increase in the surrounding fat tissue concentration, suggesting a depth of infiltration beyond the subserosa (SS). In addition, multiple enlarged lymph nodes were noted in the lesser curvature, raising the suspicion of lymph node metastases. No obvious distant metastases or peritoneal dissemination were observed. Based on these findings, the patient was diagnosed with GC, classified as clinical stage III (T3N1M0). The patient underwent laparoscopic distal gastrectomy with D2 lymph node dissection and Billroth I reconstruction without any other incurable factors. The patient recovered uneventfully and was discharged 10 days postoperatively. Pathological examination classified the tumor as stage II (pT3N0M0).

**Fig. 1:**
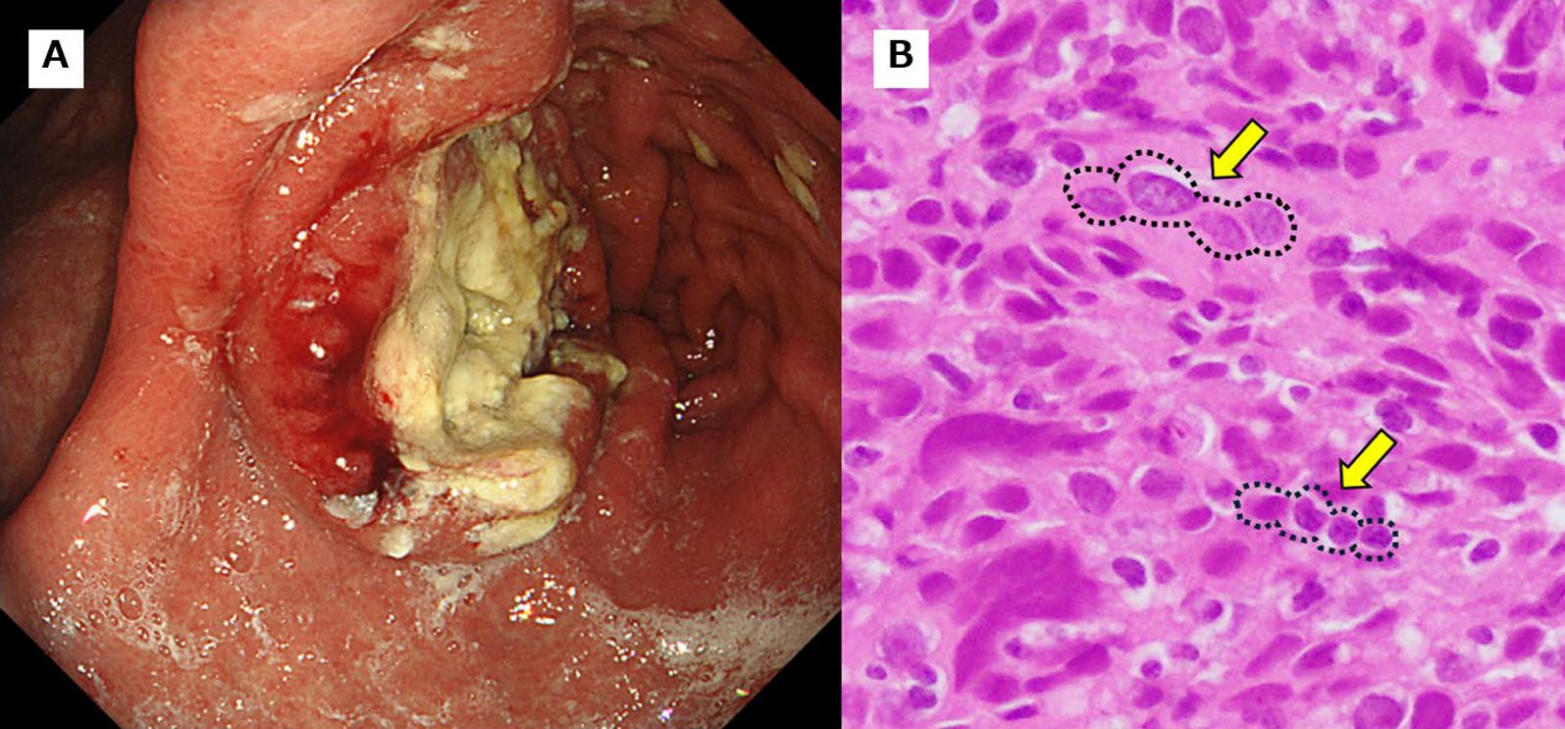
**A** Results of upper gastrointestinal endoscopy 1 year after initial surgery. Upper gastrointestinal endoscopy showing a Type 2 tumor in the efferent side of the remaining stomach. **B** Pathological findings of remaining stomach. From the biopsy of the remaining stomach tissue obtained by upper gastrointestinal endoscopy, a poorly differentiated adenocarcinoma was found histologically, characterized by the formation of solid nests and lacking glandular structures. Some tumor infiltrating lymphocytes, as indicated by arrows, were observed

**Fig. 2:**
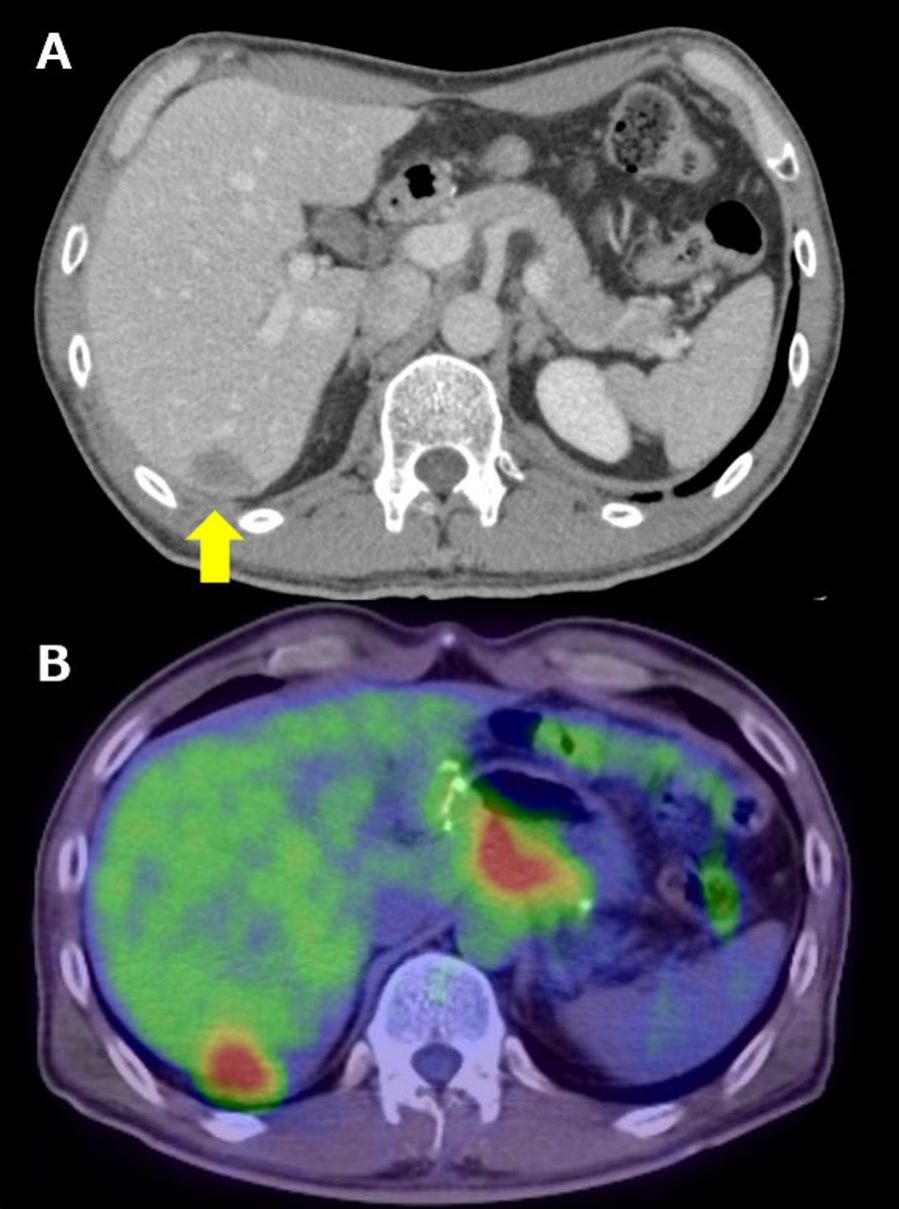
Result of CT 1 year after initial surgery. **A** CT showing a 2.5 cm low-density area in segments 6 and 7 of the liver. **B** FDG–PET performed at the same time as CT showing abnormal uptake at the site of liver metastasis

Following surgery, the patient did not receive adjuvant chemotherapy and was followed-up regularly by walk-in check-up without evidence of recurrence. However, during the first year after surgery, upper gastrointestinal endoscopy revealed a type 2 tumor on the efferent side of the remaining stomach (Fig. 1A). A biopsy was performed and adenocarcinoma was detected in the pathological examination. Some tumor infiltrating lymphocytes are also observed in Fig. 1B. Contrast-enhanced CT revealed a 2.5 cm low-density area in segments 6 and 7 of the liver (Fig. 2A). Fluorodeoxyglucose–positron emission tomography (FDG–PET) showed abnormal uptake only at the site of the liver metastasis (Fig. 2B). The patient was diagnosed with remnant gastric cancer classified as clinical stage IVB (T3N0M1(HEP)). Blood testing revealed a CEA level of 1.6 ng/ml and a CA19-9 level of under 0.1 U/ml, which still indicated no elevation of tumor markers. Histopathological examination of the biopsy specimen indicated poorly differentiated adenocarcinoma, HER2 status was negative and positive expression of PD-L1 (combined positive score [CPS] ≥5).

**Fig. 3:**
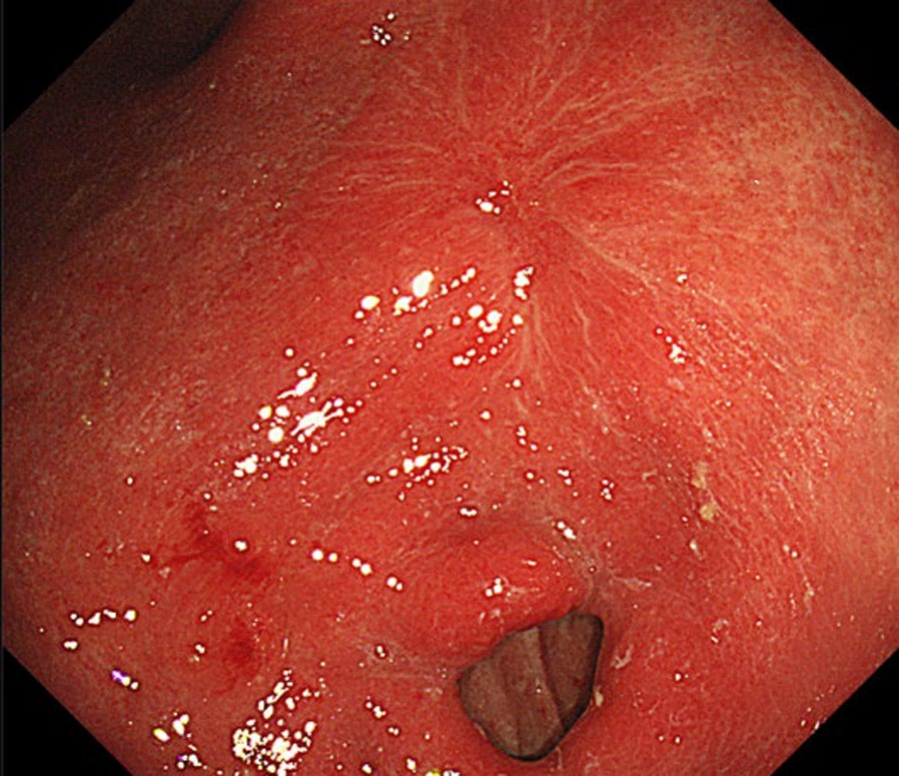
Result of upper gastrointestinal endoscopy after four courses of nivolumab combination therapy. Upper gastrointestinal endoscopy revealed a trend in tumor reduction in the gastric remnant

**Fig. 4:**
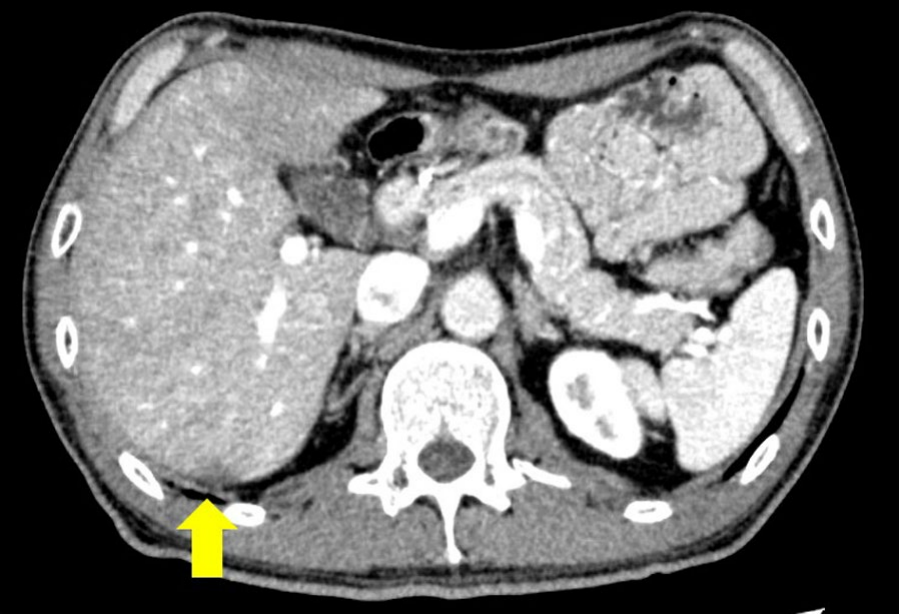
Result of CT after 4 courses of nivolumab combination therapy. CT demonstrating a reduction in the size of the liver metastasis to approximately 1 cm

The patient commenced first-line chemotherapy with a regimen comprising TS-1 (tegafur 120 mg/day for 14 days), oxaliplatin (250 mg/body/day), and nivolumab (360 mg/body/day). After completing four cycles of this therapy, upper gastrointestinal endoscopy revealed a trend of tumor reduction in the gastric remnant (Fig. 3), while contrast-enhanced CT demonstrated a reduction in the size of the liver metastasis to approximately 1 cm (Fig. 4). No new metastatic lesions were observed, and the patient's overall condition remained favorable.

**Fig. 5:**
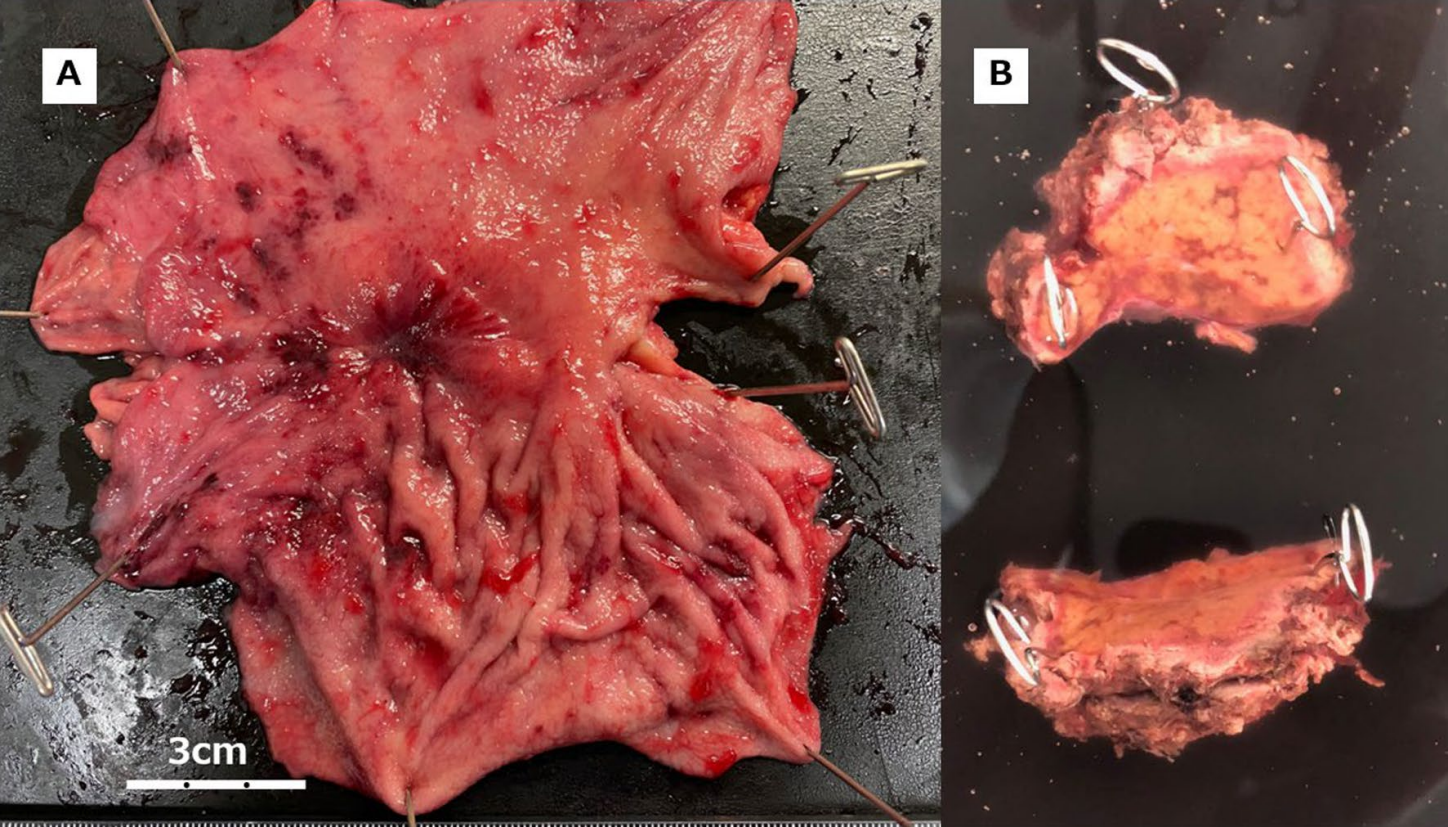
Resected remnant GC and liver with metastasis. **A** Resected remnant GC. Tumor residue was observed macroscopically, raising suspicion of malignancy. **B** Resected liver metastasis. The presence of the tumor was not clearly evident macroscopically

**Fig. 6:**
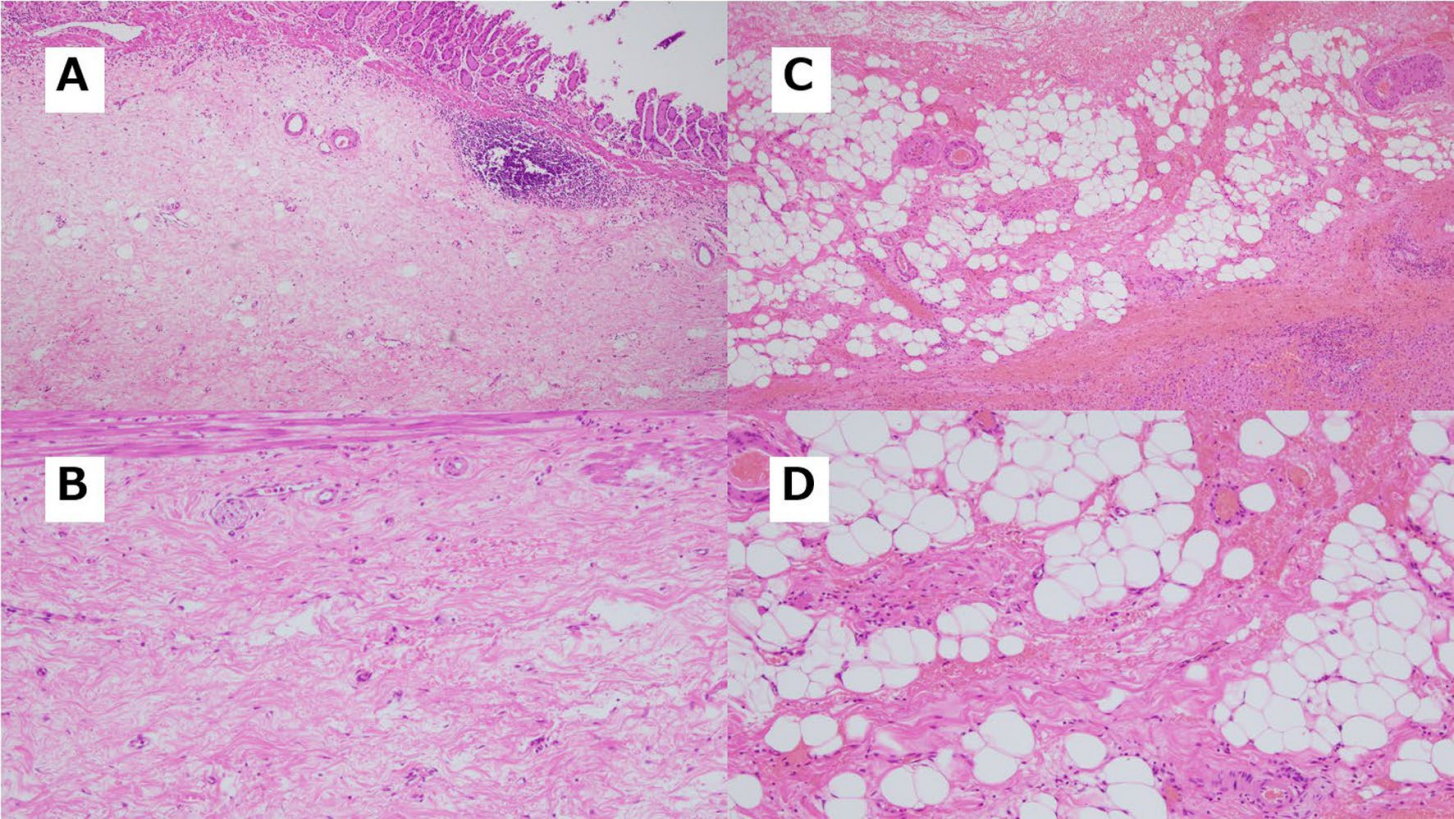
Pathological findings after conversion surgery. **A,** **B** Pathological finding of remnant GC. **A** is with low power field and **B** is with high power field. The lesion, macroscopically classified as type 3, as shown in Fig. 5A, was dissected for pathological examination. However, no poorly differentiated adenocarcinoma was found. Extensive fibrosis and scarring were observed in all sections, with no apparent malignant cells identified. Therefore, histological assessment of therapeutic effect was determined to be grade 3, indicating evidence of pCR. **C,** **D** Pathological findings of liver with metastasis. **C** is with low power field and **D** is with high power field. This demonstrates fibrous scar formation with moderate infiltration of lymphocytes and histiocytes, suggesting the disappearance of cancer cells due to preoperative treatment. No residual malignant cells were identified

Given these positive treatment outcomes and the absence of new metastatic lesions, we decided to proceed with conversion surgery. The patient underwent laparoscopic partial hepatectomy and total gastrectomy with Roux-en-Y reconstruction for remnant gastric cancer and liver metastasis. The surgical procedure lasted for 8 h and 27 min, with a blood loss of 15 ml. No significant issues were encountered during surgery, and the patient was discharged 7 days postoperatively. Figure 5A shows a specimen of resected remnant GC and Fig. 5B shows a resected liver with metastasis. Macroscopically, there were findings suggestive of malignancy, however, pathological examination of the resected specimens revealed no malignant findings at the primary tumor site or liver metastasis (Fig. 6), confirming pCR.

Postoperatively, the patient received adjuvant chemotherapy with **S-1** (tegafur 120 mg/day) for 1 year, and remained recurrence-free for 18 months following surgery.

## Discussion

GC remains the leading cause of cancer-related mortality worldwide [1]. Despite the ongoing developments in treatment strategies, patients diagnosed with stage IV GC continue to have a poor prognosis.

Advanced GC with liver metastasis is characterized by limited therapeutic options, while the poor prognosis poses a significant clinical challenge. In recent years, immunotherapy, particularly the integration of ICIs, has emerged as a promising approach for improving outcomes in this challenging patient population. Nivolumab combination chemotherapy has further been anticipated as a potentially curative treatment option for such scenarios, and is recommended as a first-line regimen in the Japanese GC guidelines [9]. Consequently, an increasing number of patients with stage IV GC are now considered eligible for conversion surgery after combination therapy. According to classifications such as that designed by Yoshida, in which categories 1 and 2 may eventually undergo conversion surgery [11], there is growing acceptance that chemotherapy for stage IV GC is undeniably associated with survival advantages. Chemotherapy for stage IV GC downsizes the tumor, increases the likelihood of radical resection, and eliminates micrometastases from cancer cells [12]. Recent studies have highlighted the prognostic value of conversion surgery [10, 13]. While the optimal timing for conversion surgery remains controversial, a variety of factors have been identified as prognostic indicators during this evaluation process. Traditional features such as primary tumor size, margin status, and number of lymph nodes may no longer be entirely applicable to cases that have achieved clinical downstaging after chemotherapy. Instead, attention has shifted to pathological grade as a potential prognostic factor. For digestive cancers, including GC, but not for stage IV cancers, Tao Wan et al. concluded that pCR correlates with favorable survival outcomes compared to non-pCR after surgery following chemotherapy [14]. Furthermore, for patients who successfully underwent surgery after chemotherapy, achieving pCR emerged as an independent prognostic factor, with a 3-year overall survival (OS) of 70.9%, surpassing the 48.8% OS of patients who did not achieve pCR after conversion surgery [15]. TRG (tumor regression grade (TRG) could also be an independent prognostic factor affecting the OS of GC patients [15]. These results suggest that even if chemotherapy is not completely effective, a greater efficacy is still associated with better prognosis. As such, treatment combining stringent chemotherapy with nivolumab could improve the prognosis of GC.

Regarding conversion surgery, achieving R0 resection, where the surgical margin is microscopically negative, is the most critical prognostic factor [11]. The results of the ongoing RENAISSANCE/AIO-FLOT (NCT02578368) phase III trial aimed to evaluate conversion surgery for gastric and gastroesophageal junction cancers with limited metastases are awaited [16].

Against this backdrop, our case of stage IV remnant GC with liver metastasis is significant, particularly in the context of the consideration of conversion surgery and achieving pCR after nivolumab combination chemotherapy. There are currently limited reports on achieving pCR after chemotherapy for stage IV GC. A PubMed search using the keywords < gastric cancer (GC) > , < Stage IV > , and < pathological CR > from the 1990s to 2023 yielded only 6 case reports [17–22], all treated with ICI, more specifically a programmed death-1 (PD-1) inhibitor combination chemotherapy (Table 1). Among these cases, including ours, the latest four were treated with ICI combination chemotherapy as the first-line regimen, whereas the others received ICI as the third or later regimen. All patients included in our study underwent conversion surgery with R0 resection and achieved a histological response grade 3, indicating eventual pCR. Our case had the longest follow-up duration among all reported patients treated with ICI as first-line therapy. In addition, unlike some patients who experienced adverse effects from ICI, our patient did not. This is noteworthy as the onset of adverse events can prompt the cessation of ICI or chemotherapy, guiding subsequent treatment steps, such as conversion surgery.

**Table 1:** Summary of previously reported cases achieving pCR after ICI combination therapy for stage IV GC

References	Years	Age	Metastasis	CPS	Number of chemotherapy regimens prior to ICI	Regimen of ICI combination therapy (Course)	Conversion surgery (approach)	Post ICI Chemotherapy	Adverse event	RFS (months)	Outcome
Zhe Zhu [17]	2023	63	Virchow LN	≥ 5	–	capecitabine, oxaliplatin, tislelizumab (8)	TG (laparoscopy)	XELOX	gastrointestinal bleeding	14	Alive
Izumo [18]	2023	82	Virchow LN	≥ 5	–	SOX, nivolumab (3)	DG + LN resection (robot assisted)	–	–	N/A	Alive
Dezhao [19]	2022	60	liver	1	–	SOX, camrelizumab, iNKT cells (2)	TG + LM (N/A)	–	Myelo-suppression	11	Alive
Toyota [20]	2022	73	liver	≥ 5	–	SOX, nivolumab (5) (+ radiation)	DG + LM (N/A)	–	–	N/A	Alive
Pan [21]	2022	45	liver, lung	N/A	3	nivolumab (5)	TG (N/A)	nivolumab	Exfoliative dermatitis eruption	28	Alive
Matsumoto [22]	2020	68	liver, lung	N/A	2	nivolumab (26)	DG (laparoscopy)	nivolumab	–	3	Alive
This case	-	54	liver	≥ 5	–	SOX, nivolumab (4)	TG + LM (laparoscopy)	S1	–	18	Alive

*TG* total gastrectomy, *DG* distal gastrectomy, *LM* liver node, *LN* lymph node, *N/A* not available

The number of cases following a favorable clinical course with ICI is expected to increase in the near future, necessitating further discussion on the merits and efficacy of ICI as a prospective treatment, along with determining the necessary duration of administration. Deciding the timing of conversion surgery remains controversial. Hence, it is imperative that more case reports and prospective studies are published to enhance our understanding and improve patient survival outcomes.

Finally, regarding postoperative treatment, there is no evidence for adjuvant chemotherapy after conversion surgery for Stage IV GC, and Japanese GC guidelines do not specify a treatment policy for such cases. Thus, we referred to the ongoing Japan Clinical Oncology Group (JCOG) study as follow.

In Japan, a randomized phase III trial, JCOG1509 called NAGISA trial [23], is currently underway to evaluate the superiority of neoadjuvant chemotherapy in locally advanced GC followed with gastrectomy and postoperative chemotherapy. In this trial, patients with cT3-4N1-3 advanced GC are divided into two groups: Group A [surgery (gastrectomy with D2 lymphadenectomy) and postoperative adjuvant chemotherapy] and Group B [neoadjuvant chemotherapy (S-1 and Oxaliplatin for 3 courses), surgery (gastrectomy with D2 lymphadenectomy) and postoperative adjuvant chemotherapy]. The aim is to compare the prognosis of both groups and to verify the effectiveness of neoadjuvant chemotherapy for advanced GC. In this trial, the regimen used for postoperative adjuvant chemotherapy consists of S-1 therapy for 1 year for ypStage 0–II cases and DS therapy for 1 year for ypStage III–IV cases.

Although this trial is not designed for stageIV GC and now ongoing, based on the fact that this case was ypStage0 after chemotherapy followed by R0 surgery, we followed the regimen of this trial, and oral S-1 monotherapy was administered for 1 year as postoperative adjuvant chemotherapy.

## Conclusion

The present case report underscores the evolving role of nivolumab combination therapy as a potential first-line treatment for patients with remnant gastric cancer and liver metastasis. The prospect of achieving pCR and conversion surgery represents a significant advancement in the management of advanced gastric cancer with distant metastases.

## Data Availability

All data generated or analyzed during this study are included in this published article.

## Abbreviations

pCR: Pathological complete response
GC: Gastric cancer
ICI: Immune checkpoint inhibitors
PD-1: Programmed death-1
MST: Median survival time
CEA: Carcinoembryonic antigen
TMB: Tumor mutation burden
MMR: Mismatch repair
CT: Computed tomography
SS: Subserosa
CPS: Combined positive score
TRG: Tumor regression grade
TG: Total gastrectomy
DG: Distal gastrectomy
LM: Liver metastasectomy
LN: Lymph node
N/A: Not available
JCOG: Japan Clinical Oncology Group
